# P04.41. Prevalence of complementary and alternative medicine use in a community-based population in Korea: a systematic review

**DOI:** 10.1186/1472-6882-12-S1-P311

**Published:** 2012-06-12

**Authors:** S Baek, H Seo, S Kim, S Choi

**Affiliations:** 1 Korea Institute of Oriental Medicine, Daejeon, Republic of Korea; 2 Department of Nursing, College of Medicine, Chosun University, Gwangju, Republic of Korea

## Purpose

The use of complementary and alternative medicine (CAM) is increasing in East Asian countries as well as in Western nations. However, information regarding the prevalence of the use of CAM in community-based populations is inconsistent and has not been systematically reviewed. This review examines the prevalence of CAM use in the Republic of Korea, focusing on (1) factors that possibly cause considerable variations in the reported prevalence, (2) the relationship between CAM use and the methodological qualities of surveys, and (3) socio-demographic factors associated with the use of CAM.

## Methods

A systematic search of electronic databases (e.g., Medline, CINAHL, Kmbase, KoreaMed, KISS, KiSTi, NDSL and OASIS) was conducted based on a predefined search strategy and selection criteria. We included cross-sectional studies that examined the Korean population in community settings and presented the percentage of CAM use as the main outcome. Data collection and assessment of the methodological quality of the selected studies were conducted by three independent reviewers.

## Results

A total of 11 studies that met our selection criteria were identified. CAM use in Korea varied from 29% to 83%. Other important findings were as follows: (1) the scope of CAM use and the taxonomies used to describe CAM modalities were inconsistent across studies, (2) recall bias, lack of representative sampling strategies, and pilot testing comprised vulnerable areas of methodological risk, and (3) demographic factors most affected by CAM use in the Republic of Korea were female gender, higher education and age.

## Conclusion

Researchers should conduct methodologically well-designed surveys of CAM use by utilizing critical quality components. The development of a specific definition of CAM and a classification of CAM modalities that reflect regional specificities are needed to conduct better comparative studies among multiple other countries.

